# (*E*)-4-[(4-Bromo­phen­yl)imino­meth­yl]-2-meth­oxy­phenol

**DOI:** 10.1107/S1600536812031704

**Published:** 2012-07-18

**Authors:** Karla Fejfarová, Michal Dušek, Sepideh Maghsodlou Rad, Aliakbar Dehno Khalaji

**Affiliations:** a Institute of Physics ASCR, v.v.i., Na Slovance 2, 182 21 Prague 8, Czech Republic; b Department of Chemistry, Faculty of Science, Golestan University, Gorgan, Iran

## Abstract

In the crystal structure of the title compound, C_14_H_12_BrNO_2_, the dihedral angle between the rings is 37.87 (10)° and the mol­ecule has an *E* conformation about the central C=N bond. In the crystal, mol­ecules are connected by inter­molecular O—H⋯N hydrogen bonds into zigzag chains running parallel to the *b* axis. The packing also features C—H⋯O inter­actions.

## Related literature

For Schiff base derivatives and related structures, see: Fejfarová *et al.* (2010*a* ▶,*b* ▶); Özek *et al.* (2009 ▶, 2010 ▶); Akkurt *et al.* (2008 ▶); Khalaji *et al.* (2007 ▶, 2009 ▶). For applications and properties of Schiff base compounds, see: da Silva *et al.* (2011 ▶); Dalapati *et al.* (2011 ▶); Sun *et al.* (2012 ▶).
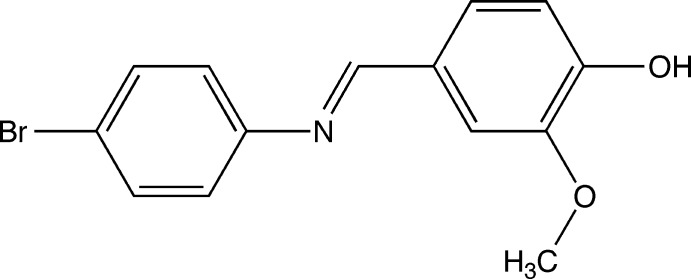


## Experimental

### 

#### Crystal data

C_14_H_12_BrNO_2_
*M* *_r_* = 306.2
Monoclinic, 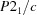

*a* = 6.5692 (3) Å
*b* = 11.4323 (4) Å
*c* = 17.5552 (9) Å
β = 97.798 (4)°
*V* = 1306.22 (10) Å^3^
*Z* = 4
Cu *K*α radiation
μ = 4.24 mm^−1^
*T* = 120 K
0.33 × 0.13 × 0.04 mm

#### Data collection

Agilent Xcalibur diffractometer with an Atlas (Gemini ultra Cu) detector
Absorption correction: multi-scan (*CrysAlis PRO*; Agilent, 2011 ▶) *T*_min_ = 0.432, *T*_max_ = 1
12474 measured reflections
2320 independent reflections
1991 reflections with *I* > 3σ(*I*)
*R*_int_ = 0.044

#### Refinement

*R*[*F* ^2^ > 3σ(*F* ^2^)] = 0.028
*wR*(*F* ^2^) = 0.067
*S* = 1.65
2320 reflections
166 parameters
1 restraint
H atoms treated by a mixture of independent and constrained refinement
Δρ_max_ = 0.27 e Å^−3^
Δρ_min_ = −0.37 e Å^−3^

### 

Data collection: *CrysAlis PRO* (Agilent, 2011 ▶); cell refinement: *CrysAlis PRO*; data reduction: *CrysAlis PRO*; program(s) used to solve structure: *SIR2002* (Burla *et al.*, 2003 ▶); program(s) used to refine structure: *JANA2006* (Petříček *et al.*, 2006 ▶); molecular graphics: *DIAMOND* (Brandenburg & Putz, 2005 ▶); software used to prepare material for publication: *JANA2006*.

## Supplementary Material

Crystal structure: contains datablock(s) global, I. DOI: 10.1107/S1600536812031704/pk2433sup1.cif

Structure factors: contains datablock(s) I. DOI: 10.1107/S1600536812031704/pk2433Isup2.hkl

Additional supplementary materials:  crystallographic information; 3D view; checkCIF report

## Figures and Tables

**Table 1:** Hydrogen-bond geometry (Å, °)

*D*—H⋯*A*	*D*—H	H⋯*A*	*D*⋯*A*	*D*—H⋯*A*
C2—H2⋯O2^i^	0.96	2.31	3.247 (3)	165
C14—H14⋯O2^ii^	0.96	2.40	3.325 (3)	163
O2—H2*o*⋯N1^iii^	0.85 (2)	1.98 (2)	2.787 (2)	158 (3)

Symmetry codes: (i) 
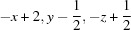
; (ii) 
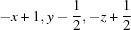
; (iii) 
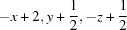
.

**Fig. 1.**
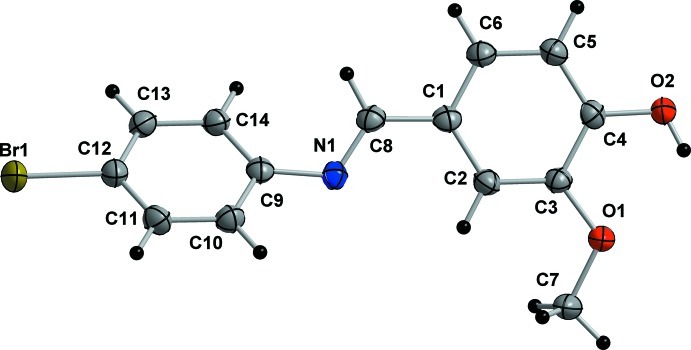
Molecular structure of (1). Displacement ellipsoids are drawn at the 50% probability level.

**Fig. 2.**
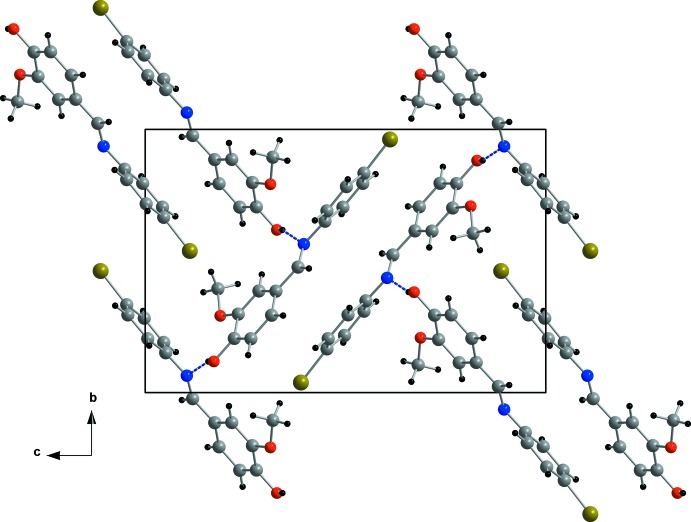
Packing of molecules in direction of a axis. Hydrogen bonds are drawn as dashed lines.

## supplementary crystallographic information

### Comment

Schiff base compounds exhibit a broad range of biological activities, including antifungal and antibacterial properties (da Silva *et al.*, 2011). They are used as anion sensors (Dalapati *et al.*, 2011) and as non-linear optics compounds (Sun *et al.*, 2012).

The present work is part of a ongoing structural study of Schiff bases (Khalaji *et al.*, 2009; Fejfarová *et al.*, 2010*a*,*b*) and we report here the structure of (*E*)-(4-hydroxy-3-methoxybenzylidene)-4-bromoaniline, (1). In the crystal, the dihedral angle between the two phenyl rings is 37.87 (10)° and the molecule has an *E* conformation about the central C=N bond. The methoxy group is slightly twisted from the attached benzene ring [C2—C3—O1—C7 = 13.8 (3)°]. The C=N and C—N bond lengths of 1.283 (3) Å and 1.419 (3) Å agree well with the corresponding distances in other Schiff bases (Akkurt *et al.*, 2008; Özek *et al.*, 2009, 2010; Khalaji *et al.*, 2007, 2009; Fejfarová *et al.*, 2010*a*,*b*).

The molecules are connected by intermolecular O—H···N hydrogen bonds, forming zigzag chains parallel to the *b* axis (Fig. 2). The crystal structure is further stabilized by intermolecular C—H···O hydrogen bonds.

### Experimental

To a stirring solution of the 4-hydroxy-3-methoxybenzaldehyde (0.2 mmol, in 5 ml of methanol) was added 4-bromoaniline (0.2 mmol) in 10 ml of methanol, and the mixture was stirred for 1 h in air at 323 K and was then left at room temperature for several days without disturbance yielding suitable crystals of (1) that subsequently were filtered off and washed with Et_2_O. Yield: 91%.

### Refinement

All H atoms bonded to carbon atoms were positioned geometrically and treated as riding on their parent atoms. The methyl H atoms were allowed to rotate freely about the adjacent C—O bonds. The hydroxyl H atoms were found in difference Fourier maps and their coordinates were refined with a restraint on the O—H bond length 0.85 Å with σ of 0.01. All hydrogen atoms were refined with thermal displacement coefficients *U*_iso_(H) set to 1.5*U*_eq_(C, O) for methyl and hydroxyl groups and to 1.2Ueq(C) for the CH and CH_2_ groups.

### Crystal data

**Table:**

C_14_H_12_BrNO_2_	*F*(000) = 616
*M**_r_* = 306.2	*D*_x_ = 1.556 Mg m^−^^3^
Monoclinic, *P*2_1_/*c*	Cu *K*α radiation, λ = 1.5418 Å
Hall symbol: -P 2ybc	Cell parameters from 6414 reflections
*a* = 6.5692 (3) Å	θ = 3.9–66.9°
*b* = 11.4323 (4) Å	µ = 4.24 mm^−^^1^
*c* = 17.5552 (9) Å	*T* = 120 K
β = 97.798 (4)°	Plate, colourless
*V* = 1306.22 (10) Å^3^	0.33 × 0.13 × 0.04 mm
*Z* = 4	

### Data collection

**Table:**

Agilent Xcalibur diffractometer with an Atlas (Gemini ultra Cu) detector	2320 independent reflections
Radiation source: Enhance Ultra (Cu) X-ray Source	1991 reflections with *I* > 3σ(*I*)
Mirror monochromator	*R*_int_ = 0.044
Detector resolution: 10.3784 pixels mm^-1^	θ_max_ = 67.0°, θ_min_ = 4.6°
Rotation method data acquisition using ω scans	*h* = −7→7
Absorption correction: multi-scan (*CrysAlis PRO*; Agilent, 2011)	*k* = −13→13
*T*_min_ = 0.432, *T*_max_ = 1	*l* = −19→20
12474 measured reflections	

### Refinement

**Table:**

Refinement on *F*^2^	45 constraints
*R*[*F*^2^ > 2σ(*F*^2^)] = 0.028	H atoms treated by a mixture of independent and constrained refinement
*wR*(*F*^2^) = 0.067	Weighting scheme based on measured s.u.'s *w* = 1/(σ^2^(*I*) + 0.0004*I*^2^)
*S* = 1.65	(Δ/σ)_max_ = 0.0003
2320 reflections	Δρ_max_ = 0.27 e Å^−^^3^
166 parameters	Δρ_min_ = −0.37 e Å^−^^3^
1 restraint	

### Special details

**Table:**

Experimental. Absorption correction: *CrysAlis PRO* (Agilent, 2011) Empirical absorption correction using spherical harmonics, implemented in SCALE3 ABSPACK scaling algorithm.
Refinement. The refinement was carried out against all reflections. The conventional *R*-factor is always based on *F*. The goodness of fit as well as the weighted *R*-factor are based on *F* and *F*^2^ for refinement carried out on *F* and *F*^2^, respectively. The threshold expression is used only for calculating *R*-factors *etc*. and it is not relevant to the choice of reflections for refinement.The program used for refinement, Jana2006, uses the weighting scheme based on the experimental expectations, see _refine_ls_weighting_details, that does not force *S* to be one. Therefore the values of *S* are usually larger than the ones from the *SHELX* program.

### Fractional atomic coordinates and isotropic or equivalent isotropic displacement parameters (Å^2^)

**Table:**

	*x*	*y*	*z*	*U*_iso_*/*U*_eq_	
Br1	0.68807 (4)	0.03813 (2)	0.611307 (17)	0.04255 (10)	
O1	1.0904 (2)	0.70826 (13)	0.18744 (10)	0.0341 (5)	
O2	0.8114 (2)	0.88161 (13)	0.17128 (9)	0.0283 (5)	
N1	0.8397 (3)	0.43584 (16)	0.39607 (10)	0.0260 (6)	
C1	0.7469 (3)	0.61449 (19)	0.32641 (12)	0.0268 (6)	
C2	0.9135 (3)	0.61379 (19)	0.28390 (13)	0.0278 (7)	
C3	0.9362 (3)	0.70156 (18)	0.23240 (13)	0.0260 (6)	
C4	0.7941 (3)	0.79425 (18)	0.22238 (13)	0.0252 (6)	
C5	0.6294 (3)	0.79520 (19)	0.26391 (13)	0.0292 (7)	
C6	0.6055 (3)	0.70607 (19)	0.31557 (13)	0.0296 (7)	
C7	1.2114 (4)	0.6054 (2)	0.18148 (15)	0.0342 (8)	
C8	0.7179 (3)	0.52315 (19)	0.38158 (12)	0.0278 (7)	
C9	0.7943 (3)	0.34873 (18)	0.44886 (12)	0.0263 (6)	
C10	0.9598 (3)	0.29543 (19)	0.49366 (13)	0.0304 (7)	
C11	0.9292 (4)	0.2049 (2)	0.54324 (13)	0.0326 (7)	
C12	0.7312 (4)	0.16602 (19)	0.54612 (13)	0.0308 (7)	
C13	0.5637 (3)	0.21731 (19)	0.50239 (13)	0.0312 (7)	
C14	0.5954 (3)	0.30931 (19)	0.45378 (13)	0.0281 (7)	
H2	1.012127	0.551422	0.290947	0.0333*	
H5	0.531306	0.857837	0.256922	0.035*	
H6	0.490709	0.70739	0.344094	0.0355*	
H7a	1.305933	0.618817	0.14521	0.0513*	
H7b	1.286592	0.587401	0.230856	0.0513*	
H7c	1.12292	0.541111	0.164378	0.0513*	
H8	0.600817	0.52848	0.408718	0.0334*	
H10	1.096928	0.321827	0.490106	0.0365*	
H11	1.043401	0.169756	0.575023	0.0391*	
H13	0.42723	0.189582	0.505637	0.0374*	
H14	0.480191	0.345928	0.423465	0.0337*	
H2o	0.930 (2)	0.882 (2)	0.1576 (16)	0.0424*	

### Atomic displacement parameters (Å^2^)

**Table:**

	*U*^11^	*U*^22^	*U*^33^	*U*^12^	*U*^13^	*U*^23^
Br1	0.04436 (17)	0.03681 (16)	0.05028 (18)	0.00744 (11)	0.02019 (12)	0.01743 (12)
O1	0.0363 (9)	0.0270 (8)	0.0426 (9)	0.0074 (6)	0.0190 (7)	0.0074 (7)
O2	0.0264 (8)	0.0245 (8)	0.0347 (9)	0.0004 (6)	0.0066 (6)	0.0055 (6)
N1	0.0294 (9)	0.0254 (9)	0.0235 (9)	−0.0014 (7)	0.0044 (8)	0.0003 (7)
C1	0.0314 (11)	0.0256 (11)	0.0237 (11)	0.0006 (9)	0.0050 (9)	−0.0045 (9)
C2	0.0310 (11)	0.0240 (11)	0.0287 (11)	0.0035 (9)	0.0054 (9)	−0.0003 (9)
C3	0.0275 (10)	0.0251 (11)	0.0261 (11)	−0.0003 (8)	0.0063 (9)	−0.0029 (9)
C4	0.0264 (10)	0.0216 (10)	0.0271 (11)	−0.0035 (8)	0.0014 (9)	−0.0031 (9)
C5	0.0294 (11)	0.0259 (11)	0.0327 (12)	0.0030 (9)	0.0062 (9)	−0.0012 (9)
C6	0.0306 (11)	0.0297 (11)	0.0299 (12)	0.0018 (9)	0.0093 (9)	−0.0022 (9)
C7	0.0317 (12)	0.0276 (12)	0.0459 (14)	0.0044 (9)	0.0146 (11)	0.0005 (10)
C8	0.0315 (11)	0.0288 (12)	0.0243 (11)	−0.0001 (9)	0.0075 (9)	−0.0043 (9)
C9	0.0321 (11)	0.0237 (11)	0.0237 (11)	0.0004 (8)	0.0066 (9)	−0.0037 (9)
C10	0.0255 (11)	0.0327 (12)	0.0331 (12)	−0.0009 (9)	0.0043 (9)	0.0011 (10)
C11	0.0317 (12)	0.0339 (13)	0.0317 (12)	0.0057 (9)	0.0025 (10)	0.0053 (10)
C12	0.0349 (12)	0.0271 (11)	0.0318 (12)	0.0024 (9)	0.0101 (10)	0.0035 (9)
C13	0.0276 (11)	0.0321 (12)	0.0356 (13)	−0.0005 (9)	0.0108 (10)	−0.0005 (10)
C14	0.0275 (11)	0.0302 (12)	0.0273 (11)	0.0032 (9)	0.0060 (9)	0.0010 (9)

### Geometric parameters (Å, º)

**Table:**

Br1—C12	1.901 (2)	C6—H6	0.96
O1—C3	1.368 (3)	C7—H7a	0.96
O1—C7	1.431 (3)	C7—H7b	0.96
O2—C4	1.358 (3)	C7—H7c	0.96
O2—H2o	0.846 (19)	C8—H8	0.96
N1—C8	1.283 (3)	C9—C10	1.393 (3)
N1—C9	1.419 (3)	C9—C14	1.396 (3)
C1—C2	1.406 (3)	C10—C11	1.385 (3)
C1—C6	1.396 (3)	C10—H10	0.96
C1—C8	1.454 (3)	C11—C12	1.382 (3)
C2—C3	1.372 (3)	C11—H11	0.96
C2—H2	0.96	C12—C13	1.384 (3)
C3—C4	1.407 (3)	C13—C14	1.388 (3)
C4—C5	1.384 (3)	C13—H13	0.96
C5—C6	1.387 (3)	C14—H14	0.96
C5—H5	0.96		
			
C3—O1—C7	117.28 (17)	H7a—C7—H7b	109.4709
C4—O2—H2o	111.1 (19)	H7a—C7—H7c	109.4715
C8—N1—C9	119.6 (2)	H7b—C7—H7c	109.4709
C2—C1—C6	118.9 (2)	N1—C8—C1	123.8 (2)
C2—C1—C8	122.1 (2)	N1—C8—H8	118.1105
C6—C1—C8	119.0 (2)	C1—C8—H8	118.1102
C1—C2—C3	120.5 (2)	N1—C9—C10	117.3 (2)
C1—C2—H2	119.7453	N1—C9—C14	123.25 (18)
C3—C2—H2	119.747	C10—C9—C14	119.3 (2)
O1—C3—C2	125.18 (19)	C9—C10—C11	120.9 (2)
O1—C3—C4	114.60 (19)	C9—C10—H10	119.5592
C2—C3—C4	120.2 (2)	C11—C10—H10	119.5589
O2—C4—C3	121.5 (2)	C10—C11—C12	118.7 (2)
O2—C4—C5	118.94 (19)	C10—C11—H11	120.6272
C3—C4—C5	119.5 (2)	C12—C11—H11	120.6256
C4—C5—C6	120.3 (2)	Br1—C12—C11	119.14 (17)
C4—C5—H5	119.8487	Br1—C12—C13	119.19 (18)
C6—C5—H5	119.849	C11—C12—C13	121.7 (2)
C1—C6—C5	120.5 (2)	C12—C13—C14	119.2 (2)
C1—C6—H6	119.7435	C12—C13—H13	120.3851
C5—C6—H6	119.7433	C14—C13—H13	120.3861
O1—C7—H7a	109.4713	C9—C14—C13	120.15 (19)
O1—C7—H7b	109.4714	C9—C14—H14	119.9259
O1—C7—H7c	109.4714	C13—C14—H14	119.925
			
C2—C3—O1—C7	13.8 (3)		

### Hydrogen-bond geometry (Å, º)

**Table:**

*D*—H···*A*	*D*—H	H···*A*	*D*···*A*	*D*—H···*A*
C2—H2···O2^i^	0.96	2.31	3.247 (3)	165
C14—H14···O2^ii^	0.96	2.40	3.325 (3)	163
O2—H2*o*···N1^iii^	0.85 (2)	1.98 (2)	2.787 (2)	158 (3)

Symmetry codes: (i) −*x*+2, *y*−1/2, −*z*+1/2; (ii) −*x*+1, *y*−1/2, −*z*+1/2; (iii) −*x*+2, *y*+1/2, −*z*+1/2.
